# Prospective ECG-Triggered Coronary CT Angiography: Clinical Value of Noise-Based Tube Current Reduction Method with Iterative Reconstruction

**DOI:** 10.1371/journal.pone.0065025

**Published:** 2013-05-31

**Authors:** Junlin Shen, Xiangying Du, Daode Guo, Lizhen Cao, Yan Gao, Qi Yang, Pengyu Li, Jiabin Liu, Kuncheng Li

**Affiliations:** Department of Radiology, Xuanwu Hospital of Capital Medical University, Beijing, China; The University of Tennessee Health Science Center, United States of America

## Abstract

**Objectives:**

To evaluate the clinical value of noise-based tube current reduction method with iterative reconstruction for obtaining consistent image quality with dose optimization in prospective electrocardiogram (ECG)-triggered coronary CT angiography (CCTA).

**Materials and Methods:**

We performed a prospective randomized study evaluating 338 patients undergoing CCTA with prospective ECG-triggering. Patients were randomly assigned to fixed tube current with filtered back projection (Group 1, n = 113), noise-based tube current with filtered back projection (Group 2, n = 109) or with iterative reconstruction (Group 3, n = 116). Tube voltage was fixed at 120 kV. Qualitative image quality was rated on a 5-point scale (1 = impaired, to 5 = excellent, with 3–5 defined as diagnostic). Image noise and signal intensity were measured; signal-to-noise ratio was calculated; radiation dose parameters were recorded. Statistical analyses included one-way analysis of variance, chi-square test, Kruskal-Wallis test and multivariable linear regression.

**Results:**

Image noise was maintained at the target value of 35HU with small interquartile range for Group 2 (35.00–35.03HU) and Group 3 (34.99–35.02HU), while from 28.73 to 37.87HU for Group 1. All images in the three groups were acceptable for diagnosis. A relative 20% and 51% reduction in effective dose for Group 2 (2.9 mSv) and Group 3 (1.8 mSv) were achieved compared with Group 1 (3.7 mSv). After adjustment for scan characteristics, iterative reconstruction was associated with 26% reduction in effective dose.

**Conclusion:**

Noise-based tube current reduction method with iterative reconstruction maintains image noise precisely at the desired level and achieves consistent image quality. Meanwhile, effective dose can be reduced by more than 50%.

## Introduction

Since the introduction of 64- or more- slice CT technology, coronary CT angiography (CCTA) has become a well-established imaging modality for the noninvasive assessment of coronary artery disease [1]. With the constantly increasing availability of CCTA capable scanners worldwide, the number of examinations is likely to show further substantial increase. However, high radiation dose with conventional retrospective (electrocardiogram) ECG-gating draw much attention because of the usage of slow helical pitches and the x-ray beam being turned on throughout the cardiac cycle. To decrease the radiation dose, ECG controlled tube current modulation was developed. Despite this improvement, retrospective ECG-gating still leaves patients exposed to substantial radiation dose (8–19 mSv) [2], [3].

Prospective ECG-triggering, which combines step-and-shoot axial data acquisition and an incrementally moving table with adaptive ECG-triggering, represents the most effective approach with significant reduction of radiation dose by 60–80% when compared with retrospective ECG-gating [4], [5], [6]. However, despite the dose advantages of prospective ECG-triggering, dose reduction remains limited by traditional filtered back projection (FBP) reconstruction method, which produces a noticeable increase in image noise in the case of excessive reduction of radiation exposure [7].

Iterative reconstruction (IR) has been introduced to address these shortcomings of FBP [8]. By iteratively comparing each synthesized forward projection to the actual measurements and modeling system statistics, IR has the potential to selectively reduce image noise, which may permit preserved image quality with reduced tube current [9].

However, neither prospective ECG-triggering nor IR can compensate for the different chest attenuations among different patients. Therefore, individual dose optimization would be preferred. Noise-based tube current reduction method reflects the chest attenuation of each patient and can be used to optimize radiation dose individually [10].

The combination of noise-based tube current reduction method with IR can further reduce radiation dose while achieving consistent image quality [12]. Nevertheless, to the best of our knowledge, noise-based tube current reduction method with iterative reconstruction has not yet been performed with prospective ECG-triggering in a large patient cohort.

Hence, the primary aim of this prospective randomized study was to evaluate the clinical value of the noise-based tube current reduction method with iterative reconstruction for obtaining consistent image quality with dose optimization in prospective ECG-triggered CCTA.

## Materials and Methods

### Participants

**Figure 1:**
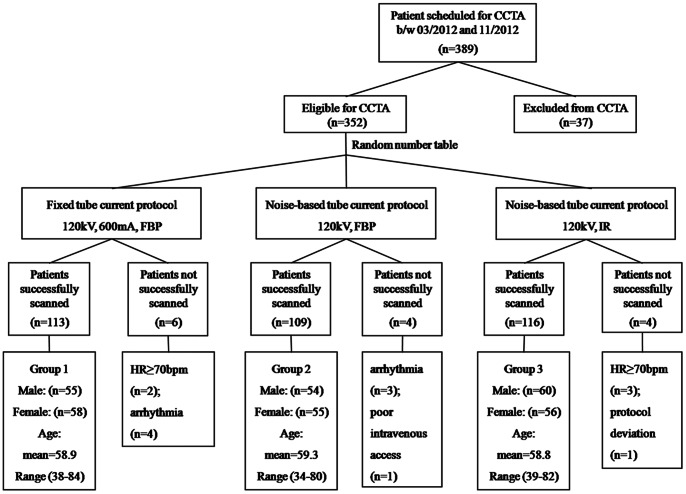
Flow chart of patient inclusion. CCTA: Coronary CT Angiography; FBP: Filtered Back Projection; IR: Iterative Reconstruction; HR: Heart Rate.

389 consecutive patients scheduled for CCTA were prospectively recruited between March 2012 and November 2012. Patients received oral β-blocker (metoprolol tartrate, 25–50 mg) if needed to achieve heart rates no more than 70 bpm. Patients with heart rate more than 70 bpm even after medications, heart rate variability more than 5 bpm or frequent ectopy were excluded from this study. Patients with known contradiction to iodinated contrast agent, inability to sustain a breath hold in the allotted time, acute coronary syndrome, heart failure and pregnant and lactating women were also excluded. Thus, 37 patients were excluded, and 352 patients were eligible for prospective ECG-triggered CCTA. These 352 eligible patients were randomly assigned into fixed tube current (Group 1), noise-based tube current with FBP (Group 2) or with IR (Group 3) by random number table. Of the 352 eligible patients, 14 patients were excluded (5 patients were excluded for heart rate more than 70 bpm during scan; 7 patients were excluded for arrhythmia during exam; and 1 patient each was excluded due to poor intravenous access and protocol deviation). A flow chart (Figure 1) illustrates the patient inclusion in our study. The study protocol had been approved by the local ethics committee of Xuanwu Hospital, and signed informed consent was obtained after the nature of the study had been explained to each patient.

### Image Acquisition

All CCTA examinations were performed on a Discovery CT750 HD scanner (GE Healthcare, Milwaukee, Wisconsin, USA) with the prospective ECG-triggering according to a commercially available protocol (snapshot pulse, GE Healthcare, Milwaukee, Wisconsin, USA). The scan sequences included a scout scanogram, a low dose calcium-scoring scan, a test-bolus (TB) scan, and a CCTA scan. The minimal z-axis coverage necessary to cover the entire coronary arteries was determined by calcium-scoring scan with the goal of minimizing radiation exposure.

Prior to each CCTA scan, TB scan was obtained every 2 s for 30 s after administration of the test bolus of 20 ml iodinated contrast (Ultravist 370; Schering, Berlin, Germany) at 5 ml/s. The timing for CCTA acquisition was determined by adding 4 s to the time of peak contrast enhancement in the center of aortic root at the level of the left main artery. The TB scan parameters were as following: 1×5 mm collimation, axial scan mode, 100 kV, 60 mA, 1.0 s rotation speed, display field of view (DFOV) of 250 mm, 512×512 matrix size, and slice thickness of 5 mm.

All scan parameters except the tube current were kept constant among the three different CCTA protocols, including 120 kV tube voltage, 64×0.625 mm collimation, 0.35 s rotation speed, DFOV of 250 mm, 512×512 matrix size, and 0.625 mm slice thickness. 60 ml of the iodinated contrast was injected followed by 40 ml saline at a rate of 5 ml/s for CCTA scan. All images were reconstructed using the same reconstruction kernel (standard). Three or four scan blocks per examination with 5 mm overlapping between blocks that can cover either 105 mm or 140 mm in the z-axis were available in our institution. A widening of the data acquisition window with the prospective scanning, also known as “padding”, was manually adjusted depending on the heart rate just before CCTA scan. In this study, 50 ms padding was used in patients with heart rates less than 65 bpm with a targeted cardiac phase at 75% of the R-R interval, while 180 ms padding in patients with heart rates between 65–70 bpm with a targeted cardiac phase at 60% of the R-R interval. After each scan, additional retrospective reconstructions at different cardiac phases were performed for patients using 180 ms padding. Specifically, 70–80% of the R-R interval (mid-diastole) was selected for heart rates less than 65 bpm during scan, while 30–45% of the R-R interval (end-systole) for heart rates between 65–70 bpm. 0 padding was not used even in patients with heart rates less than 65 bpm before CCTA scan in case of heart rate variability.

Tube current was fixed at 600 mA for all patients in Group 1, and was patient-depended on the noise of test bolus image in Group 2 and Group 3. For CCTA data reconstruction, FBP was applied for Group 1 and Group 2; and IR was applied for Group 3. IR applied in this study was ASIR (Adaptive Statistical Iterative Reconstruction; GE Healthcare, Milwaukee, Wisconsin, USA), which was performed from both the projection data and image data. Only a limited number of iterations were required to complete an entire analysis for ASIR, so the average reconstruction time was approximately 40–60% longer with the ASIR than with the standard FBP. In our study, IR represented a composite of 40% ASIR and 60% FBP [11].

The noise-based tube current reduction method employed in this study was on the basis of our prior investigations and was used to calculate the required tube current to obtain the desired CCTA noise (35HU) according to the TB noise measurement [12] (Table 1). The TB image noise measurement was performed by placing a circular region of interest (ROI) of about 100 mm^2^ in the center of aortic root on TB image. Since not all tube current settings were available, we chose the tube current closest to the suggested value.

**Table 1:** [**12**] **.** Noise-based tube current modulation formulas regarding SD_TB_ and mA_CCTA_ *.

Group	SD_TB_ (HU)	mA_CCTA_ (mA)	p Value	95% CI	r Value
Fixed tube current with FBP (Group 1)	19.48 (17.07–22.72)	600	–	–	–
Noise-based tube current with FBP (Group 2)	19.00 (16.88–21.71)	600×[(9.550+1.177SD_TB_)/35]^2^	<0.001	1.006–1.347	0.808
Noise-based tube current with IR (Group 3)	19.07 (16.40–22.21)	600×[(8.365+0.856SD_TB_)/35]^2^	<0.001	0.722–0.989	0.786

Note: SD_TB_: timing bolus noise; mA_CCTA_: tube current of CCTA; 95% CI: 95% confidence interval.

* With the noise-based tube current reduction method, the required mA_CCTA_ to obtain desired image noise level (35HU) for CCTA image can be obtained from SD_TB_ on test bolus image. SD_TB_ are median with interquartile range (IQR).

### Subjective Image Quality

Two independent readers interpreted all studies; a third independent reader achieved consensus for data with discordance. The readers had 4, 5, and 10years of experience, respectively. CCTA images were reviewed using dedicated workstations (AW 4.5 Advantage; GE Healthcare, Milwaukee, Wisconsin, USA). All images were anonymized and analyzed in random order to avoid any bias. Axial source slices and curved multi-planar reformations were assessed for each of the four main arteries (left main, left anterior descending, left circumflex and right coronary artery) in each patient. All segments with 1.5 mm or greater in diameter were evaluated. Signal intensity at the aortic root was chosen as the new window center whereas the window width was arbitrarily defined between +1000HU and +2000HU.

**Figure 2:**
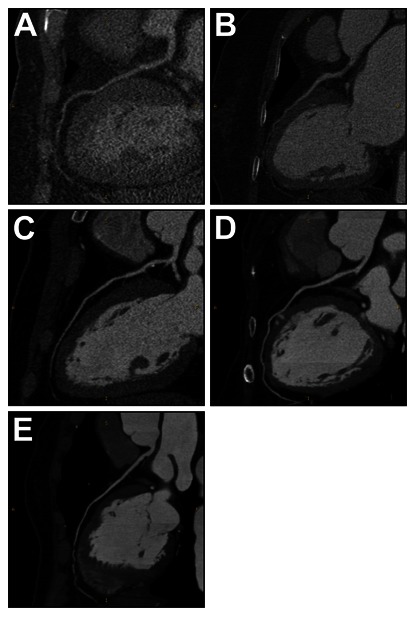
Examples of different image quality scores. Representative examples for different image quality scores. The examples show curved multiplanar reformations of left descending arteries of different patients. (a) impaired image quality, (b) reduced image quality, (c) adequate image quality, (d) good image quality, (e) excellent image quality.

Overall image quality was subjectively rated on a 5-point scale assigned on the basis of the worst scored artery as follows [11] (Figure 2 A–E): 1 = impaired image quality limited by excessive lumen noise and poor vessel wall definition or poor lumen attenuation, unacceptable for diagnosis; 2 = reduced image quality with massive lumen noise and poor vessel wall definition or low lumen attenuation, diagnosis acceptable only under limited conditions; 3 = adequate image quality with moderate lumen noise and minimal limitation of vessel wall definition, acceptable for diagnosis; 4 = good image quality with minimal lumen noise and well maintained vessel wall definition, fully acceptable for diagnosis; and 5 = excellent image quality with clear vessel wall definition and limited perceptible lumen noise, fully acceptable for diagnosis. Diagnostic image quality was considered to have been achieved for images with a score of 3 or higher. Segments with stent graft and extensive calcifications were excluded from analysis. The readers were instructed to ignore motion artifact and stair-step artifact.

### Objective Image Quality

Signal intensity, image noise and signal-to-noise ratio were quantified as objective image quality parameters. All quantitative measurements were performed on axial source CCTA images by placing a circular ROI of about 100 mm^2^ in the center of aortic root at the level of the left main artery. Signal intensity was derived from the mean CT attenuation value. Image noise was defined as the standard deviation (SD) of the CT attenuation value. Signal-to-noise ratio was calculated as the mean CT attenuation value divided by the image noise. All objective image quality was measured by one experienced reader (9years of experience).

### Radiation Dose

To estimate CT radiation dose, we recorded the volume CT dose index (CTDIvol) and the dose-length product (DLP) from the scan protocol generated by the CT system after each CCTA study. The effective radiation dose was derived from the product of the DLP and a conversion coefficient of 0.014 mSv/(mGy·cm) proposed by the European Working Group for Guidelines on Quality Criteria in CT [13] and endorsed by the American Association of Physicists in Medicine [14].

### Statistical Analysis

Quantitative variables were expressed as mean ±SD and median with interquartile range (IQR) as appropriate; categorical variables were expressed as counts (or proportions in percent). Ordinal variables were also given in means ±SD.

Comparisons between groups were performed using the one-way analysis of variance for continuous variables with normal distributions, and Kruskal-Wallis test for continuous variables with non-normal distributions. The chi-square test was used for categorical variables. Inter-observer agreement for the subjective image quality was estimate using kappa statistic.

To identify variables associated with the effective dose, multivariable linear regression models were used that respectively assessed patient characteristics only or scan characteristics only. The separate models were used because of the intrinsic interaction between patient and scan variables. The stepwise regression was used with an entry criterion of p value less than 0.05 and a removal criterion of p value more than 0.01. Patient characteristics include age, gender, heart rate and body mass index (BMI); scan characteristics included padding duration (50 ms versus 180 ms), scan range (105 mm versus 140 mm) or (3 scan blocks versus 4 scan blocks), and reconstruction algorithm (IR versus FBP). To avoid over-fitting of the multivariable models, we entered only variables with p value less than 0.10 in the univariate analysis. Because of the non-normality of the distribution of effective dose, the dose in millisieverts was converted to the logarithm of millisieverts to obtain a normal distribution for regression analysis.

All analyses were performed with SPSS software (18.0, SPSS Inc, Chicago, ?). A two-tailed p value of less than 0.05 was deemed statistically significant difference.

## Results

### Patient and Scan Characteristics

A total of 338 patients were successfully scanned without adverse events (Group 1∶55 male, 58 female, ages from 38 to 84 with mean of 58.9years, BMI from 18.0 to 41.5 with mean of 25.5 kg/m^2^; Group 2∶54 male, 55 female, ages from 34 to 80 with mean of 59.3years, BMI from 17.8 to 34.3 with mean of 25.2 kg/m^2^; Group 3∶60 male, 56 female, ages from 39 to 82 with mean of 58.8years, BMI from 17.5 to 33.8 with mean of 25.4 kg/m^2^). Table 2 contains a summary of patient and scan characteristics among 3 groups. Patient and scan characteristics were similar among 3 groups, except for the tube current, which was lowest for Group 3 (median, 300 mA; IQR, 255–379 mA) and highest for Group 1 (600 mA).

**Table 2:** Patient and scan characteristics.

Characteristics	Fixed tube current with FBP (Group 1)	Noise-based tube current with FBP (Group 2)	Noise-based tube current with IR (Group 3)	p value
N	113	109	116	
Age, yrs	58.9±9.0	59.3±9.4	58.8±8.8	0.894
Male sex	48.7 (55/113)	49.5 (54/109)	51.7 (60/116)	0.893
Heart rate*, bpm	56.8±6.5	56.2±5.7	56.6±5.8	0.752
Height, m	1.65±0.08	1.65±0.08	1.67±0.07	0.204
Weight, kg	69.4±11.4	69.0±11.8	70.5±10.4	0.598
Body mass index, kg/m^2^	25.5±3.5	25.2±3.0	25.4±3.1	0.832
Tube current, mA	600	505 (418–598)	300 (255–379)	<0.001
Padding duration, ms				0.312
50	78.8 (89/113)	86.2 (94/109)	80.2 (93/116)	
180	21.2 (24/113)	13.8 (15/109)	19.8 (23/116)	
Scan range, mm				0.770
139.38	69.0 (78/113)	73.4 (80/109)	70.7 (82/116)	
104.38	31.0 (35/113)	26.6 (29/109)	29.3 (34/116)	

Note: Data are mean±standard deviation (SD), median with interquartile range (IQR) and percentage with raw data in parentheses.

* Heart rate is mean heart rate during scanning.

### Subjective and Objective Image Quality

**Figure 3:**
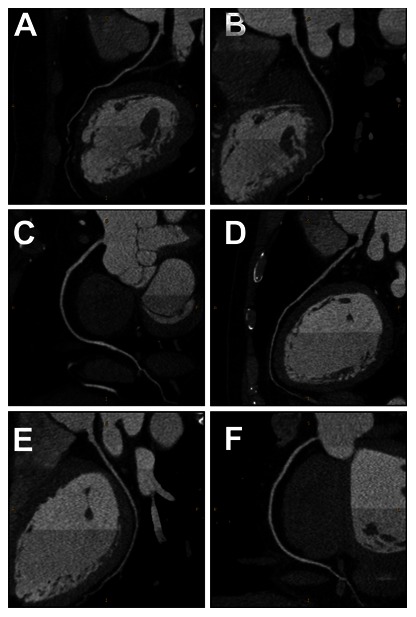
Representative CCTA demonstrating image quality with different scan protocols. 57year old woman with body mass index of 26.49 kg/m^2^ (a−c) and 42year old man with body mass index of 22.41 kg/m^2^ (d−f). Curved multiplanar reformations of left descending arteries (a, d), left circumflex arteries (b, e), and right coronary arteries (c, f) of two patients demonstrating the same noise level (35HU) and qualitative image quality (score 3) obtained by the noise-based tube current with filtered back projection (328 mA, 120 kV, 2.02 mSv; a−c) and with iterative reconstruction (195 mA, 120 kV, 1.19 mSv; d-f), respectively.

Inter-observer agreement for image quality between the 2 readers was good (κ = 0.74). The mean image quality score was 3.42±0.69 for Group 1, which was significantly greater than Group 2 (3.13±0.34, p<0.05) and Group 3 (3.09±0.29, p<0.05) (Table 3). However, both Group 2 and Group 3 scored diagnostic image quality and nearly equivalent image quality score was found between the two groups (Figure 3 A-F). In addition, the standard deviations of the image quality score in Group 2 and Group 3 were smaller than that of Group 1, reflecting more consistent image quality in Group 2 and Group 3.

**Table 3:** Image quality and radiation exposure.

Characteristics	Fixed tube current with FBP (Group 1)	Noise-based tube current with FBP (Group 2)	Noise-based tube current with IR (Group 3)	p value
Image quality score	3.42±0.69	3.13±0.34	3.09±0.29	<0.001
Signal intensity, HU	390±71	405±73	381±65	0.034
Image noise, HU	32.90 (28.73–37.87)	35.01 (35.00–35.03)	35.01 (34.99–35.02)	<0.001
Signal-to-noise ratio	12.1±3.5	11.6±2.1	10.9±1.9	0.003
CTDIvol, mGy	18.7 (18.7–18.7)	16.4 (13.3–20.1)	10.0 (8.1–13.4)	<0.001
DLP, mGy·cm	262 (196–262)	209 (176–269)	129 (108–167)	<0.001
Effective dose, mSv	3.7 (2.8–3.7)	2.9 (2.5–3.8)	1.8 (1.5–2.3)	<0.001

Note: CTDIvol: volume CT dose index; DLP: dose-length product.

Data are mean±standard deviation (SD), median with interquartile range (IQR) in parentheses.

Quantitative analysis demonstrated that significant difference in signal intensity was observed between Group 2 (405±73HU) and Group 3 (381±65HU). However, the decreased signal intensity was not obvious for Group 3 (only by 5.93%).

The image noise was significantly higher for both Group 2 (median, 35.01HU; IQR, 35.00–35.03HU) and Group 3 (median, 35.01HU; IQR, 34.99–35.02HU), when compared to Group 1 (median, 32.90HU; IQR, 28.73–37.87HU). However, the median image noise was precisely maintained at the target value of 35HU for both Group 2 and Group 3. In addition, the IQR of the image noise in Group 2 and Group 3 was much smaller than that of Group 1, reflecting more consistent image noise obtained in Group 2 and Group 3.

Signal-to-noise ratio was significantly higher for Group 1 (12.1±3.5), when compared to Group 3 (10.9±1.9; p = 0.002). However, Group 2 and Group 3 were comparable regarding signal-to-noise ratio (p = 0.160). Due to higher signal intensity for Group 2, no significant difference of signal-to-noise ratio was found between Group 1 and Group 2 (p = 0.432).

### Radiation Dose Estimates

The radiation dose parameters under different CCTA protocols are summarized in Table 3. Using the fixed tube current for Group 1, the effective dose was 3.7 mSv (IQR, 2.8–3.7 mSv). The use of the noise-based tube current with FBP for Group 2 resulted in a significant reduction in the effective dose to 2.9 mSv (IQR, 2.5–3.8 mSv, p<0.05), corresponding to a 20% dose reduction.

The use of the noise-based tube current with IR for Group 3 further reduced the effective dose by 38% to 1.8 mSv (IQR, 1.5–2.3 mSv, p<0.05). When compared with Group 1, an overall radiation dose reduction of 51% was achieved.

The univariate and multivariate analysis of patient and scan characteristics associated with effective dose are provided in Table 4. In the univariate analysis, all patient and scan variables except age and gender demonstrated a significant association with effective dose and were entered into the multivariable linear regression analysis. In the multivariable analysis, IR versus FBP (25.5%; 95% Cl, 22.3% to 27.6%; p<0.001), 50 ms versus 180 ms of padding (24.7%; 95% Cl, 22.1% to 27.4%; p<0.001), 105 mm versus 140 mm of scan range (7.2%; 95% Cl, 4.9% to 9.4%; p<0.001), heart rate (7%; 95% Cl, 4% to 10%; p<0.001) and BMI (1.2%; 95% Cl, 0.6% to 1.8%; p<0.001) were independently associated with the reduced effective dose.

**Table 4:** Patient and scan characteristics associated with effective dose*.

	Univariate Analysis		Multivariate Analysis	
	% Effects (95% CI)	p value	% Effects (95% CI)	p value
Patient characteristics				
Age, 1year increase	−0.1 (−0.3–0.1)	0.239	NA	NA
Gender, male vs female	−2.1 (−6–1.8)	0.288	NA	NA
Heart rate, 10beats/min increase	7 (4–10)	<0.001	7 (4–10)	<0.001
Body mass index, 1 kg/m^2^ increase	1.2 (0.6–1.8)	<0.001	1.2 (0.6–1.8)	<0.001
Scan characteristics				
Padding duration(ms), 50 vs 180	23.4 (19.1–27.8)	<0.001	24.7 (22.1–27.4)	<0.001
Scan range(mm), 105 vs 140	6.2 (2.0–10.5)	0.004	7.2 (4.9–9.4)	<0.001
Reconstruction Algorithm, IR vs FBP	24.9 (21.8–28.0)	<0.001	25.5 (23.3–27.6)	<0.001

Note: CI: confidence interval; NA: not applicable.

* Patient and scan characteristics associated with radiation dose are presented as % change of the effective dose (mSv).

## Discussion

In this prospective randomized study, we compared protocols with and without noise-based tube current reduction method and with and without IR concerning image quality and radiation exposure. We found that the noise-based tube current reduction method reduced the effective dose by 20%, compared with the fixed tube current protocol while maintaining clinically acceptable images with more consistent image noise (35HU). The effective dose was further reduced by 38% with IR algorithm. These findings illustrated the capability of a substantial reduction in radiation exposure by combining a noise-based tube current reduction method with IR in prospective ECG-triggered CCTA.

### Prospective ECG-triggered CCTA

A worldwide radiation dose survey indicated that CCTA scan was associated with a median dose of 12 mSv [15]. The increasing number of CCTA examinations has led to concerns regarding the risk of malignancies induced by the application of medical ionizing radiation. What’s more, high radiation dose with retrospective ECG-gating draws much attention because of the usage of slow helical pitches and the x-ray beam being turned on throughout the cardiac cycle.

Prospective ECG-triggering is performed in a non-helical way with the acquisition of a series of axial images instead of volumetric data. Thus, radiation is administrated only at the pre-defined cardiac phases rather than throughout the entire cardiac cycle, which may reduce radiation exposure by approximately 60–80% compared with the conventional retrospective ECG-gating [4], [5], [6]. A widening of the data acquisition window with prospective ECG-triggering, also known as “padding”, was recommended for all patients in our study. For low heart rate (less than 65 bmp) before CCTA scan, a narrower padding (50 mm) was used in prevention of heart rate varies during exam [16]. But if heart rate was high (more than 65 bpm) before exam, a wider padding (180 ms) was generally used. Then, additional reconstructions at different cardiac phases were obtained to find a cardiac phase with less cardiac motion. Therefore, application of padding helps to generate images with less motion artifacts in patients with higher heart rate or apparent heart rate variability. However, this leads to an increase of effective dose by up to 42% when compared to that without padding groups [17]. Our multi-linear regression analysis demonstrated that the use of a wider padding (180 ms) was associated with a considerable (24.7%) increase of effective dose while adjusting for other scan characteristics. Thus, a low and regular heart rate is necessary for reducing dose in prospective ECG-triggering. In addition, the use of a shorter scan range of 105 mm (3 scan blocks) in our study population was associated with a decrease of effective dose of approximately 6.2%. Instead of rigidly scanning from the carina to diaphragm, scan range should be adjusted individually according to a prior performed low-dose scan (calcium scoring) [15].

### Noise-Based Tube Current Reduction Method

In CCTA with a fixed tube current (600 mA), either radiaton exposure would have been excessive for small patients to obtain aesthetic image quality or inadequate for large patients to produce poor image quality. Thus, the adjustment of tube current individually is essential for radiation dose optimization. BMI is the most commonly used parameter to determine tube current in clinical CCTA [18]. However, this method sometimes lacks precision and accuracy due to variation of the chest attenuation among patients [19]. In our study, BMI had a small effect (1.2%) on effective dose. The noise-based tube current reduction method directly reflects the chest attenuation in each patient, and more accurate tube current selection and dose optimization can be achieved for CCTA [10]. Meanwhile, image noise could be precisely maintained at the desired level and more consistent image quality could be obtained across the patient populations. In line with our prior study [12], image noise was well kept at 35HU with small amount of variability [(IQR, 35.00–35.03HU) with FBP and (IQR, 34.99–35.02HU) with IR], which leads to more uniform qualitative image quality than that with a fixed tube current. Tube current modulation dose not necessarily mean tube current reduction in all cases, and in some cases (patients with high BMI) a higher tube current are required [20]. However, it does reduce the overall tube current while imparting a statistically significant reduction in radiation exposure for the entire patient population. Our results show that effective dose was reduced by 20% for protocol using noise-based tube current compared with fixed tube current with the same FBP algorithm, which was higher than the percentage of dose reduction (14%) in our previous study [12]. The higher relative dose reducing may be due to the higher percent of cases with 180 ms padding in the fixed tube current group in this study (21.2% versus 13.8%) making the baseline slightly higher than the previous study.

### Iterative Reconstruction Algorithm

In consistence with the ERSIR study [21], our study demonstrated that IR had a leading association (approximately 26%) with effective dose while adjusting for other scan characteristics. By iterative comparison of each synthesized forward projections to the actual measurements and modeling system statistics (photon statistics and image noise), IR is known for the potential to selectively reduce image noise without compromising signal intensity and spatial resolution [9]. The noise reduction properties of IR may permit the use of lower tube current with stable image noise and quality when compared with FBP using higher tube current, despite the decreased number of photons. Another advantage of IR is that it is supplemental to other radiation reduction techniques, and thus it is not necessary to compromise the choice of protocol. Our study showed that the noise-based tube current reduction method with IR reduced tube current by 40.59%, leading to a 38% decrease in effective dose without compromising image quality compared with that using FBP. This was similar to the finding of Leipsic et al. [11], who reported that noise reduction properties of IR (40%ASIR) would theoretically permit a tube current reduction of approximately 30–40%, resulting in a proportional decrease in the effective dose without altering image noise and thus image quality. In our present study, the noise-based tube current reduction method with IR reduced 51% effective dose with the median effective dose of 1.8 mSv compared with the fixed tube current protocol, which was higher than the relative dose reduction (41%) in our previous study [12]. The higher relative dose reducing in this study may also be due to the higher percentage of cases with 180 ms padding in the fixed tube current group in the current study (21.2% versus 19.8%). Since sequential scan mode performed for all patients and fixed tube current (600 mA) used in the control group in our study, higher effective dose reduction (51% versus 44%) was achieved than the ERSIR study [21]. Another study by Hou et al. [22] achieved a higher reduction in effective dose (63%) with IR (1.2 mSv) compared with FBP (3.2 mSv). This might be related to the different scanner (256-slice muti-detecter CT) and different type of IR technique (iDose, Philips Healthcare) employed in their study. Although in their study no significant difference in objective image noise and image quality score was found between the IR and FBP groups, the standard deviation for the objective image noise was relatively large in both groups. On contrast, in our stuy, image noise was precisely maintained at the disired level (35HU) and more uniform image quality was achieved.

There were several limitations in our study. First, patient inclusion was limited to those with heart rates lower than 70 bmp because of the temporal resolution of the 64-slice single-source CT system. However, with careful patient selection and screening and with effective use of β-blocker for heart rate control, prospective ECG-triggering can be used in a high percentage of CCTA exams. Second, radiation dose data presented here were for the CCTA scan only, the examination also included scout scanogram, calcium-scoring scan and TB scan, which added an additional 0.73 mSv (IQR, 0.67–0.78 mSv) to the exam. However, these pre-control scans are necessary for CCTA and present a smaller fraction of the total dose. Third, the IR algorithm used in our study represents a modified and limited form of IR. More advanced IR techniques can further improve image quality with substantial decreased image noise, allowing more aggressive reduction in radiation exposure [23]. However, high computational cost and long reconstruction time remains a barrier for the complete IR in daily clinical application.

### Conclusions

Noise-based tube current reduction method with IR maintains image noise precisely at the desired level individually and achieves consistent image quality across the entire patient population. In addition, with the former technique, overall radiation dose reduction is up to 51% but only for the patients who qualified. Based on our prospective randomized study, we recommend the use of the noise-based tube current reduction method in prospective ECG-triggered CCTA for patients with heart rates less than 70 bpm.
